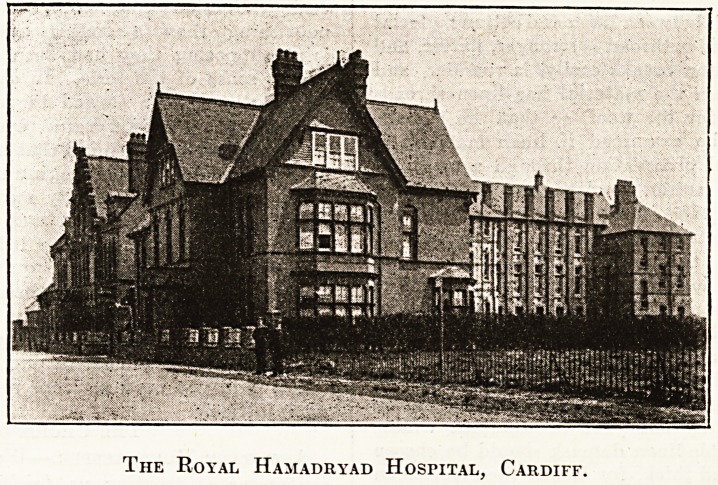# A Private Visit to the Hamadryad Seamen's Hospital

**Published:** 1912-07-06

**Authors:** 


### A Private Visit to the Hamadryad Seamen's Hospital.

On Wednesday evening last week, their Majesties the King and Queen paid a private visit to the Royal Hamadryad Seamen's Hospital, Cardiff, the only hospital in England or Wales apart from the "Dreadnought" existing purely for the treatment of seamen.

According to the instructions received, the inside of the hospital had not been decorated, with the exception of a strip of red carpet which was laid down from the entrance to the first ward, as their Majesties' desire was to inspect the wards in their usual working order.

The motor-car containing their Majesties drew up at the hospital entrance about 6 p.m. amidst scenes of great enthusiasm amongst the Boy Scouts lining the route and the crowds which had assembled to catch a glimpse of the Royal party in passing. The weather fortunately was perfect, and many friends of the institution had been accommodated with seats on the lawn in front of the hospital, where an excellent view of the King's arrival and departure was obtained.

On their arrival the King and Queen were received by the Lord Mayor, Sir John Wesley Courtis, in the absence of Lord Merthyr, the President of the Institution, who presented to their Majesties Mr. John Moore, Vice-President and Chairman of the Executive Committee, Mr. W. Jones, Vice-Chairman, Mr. J. W. Tatem, a patron of the hospital, and Dr. Whelan, the Medical Superintendent. Amongst those present were the following gentlemen of the committee with their ladies: Mr. T. M. Heywood, Mr. J. W. Tatem, Mr. E. Lowder Downing, Mr. E. Handcock, Mr. David Robertson, Mr. S. Thomas, J.P., and Dr. D. A. Fitzgerald. Mr. Herbert Whitaker, secretary of the hospital, and Miss Maclean, a regular visitor, were also present.

Mr. John Moore presented the Queen with a copy of the last annual report bound in blue morocco and gilt, which her Majesty was graciously pleased to accept. The Royal party then proceeded down the corridor to the first ward, which contains the surgical cases. Immediately on the left were two Frenchmen, one suffering from appendicitis and the other from a crushed hand. The King and Queen conversed with them for some time in their native tongue, much to their delight, and a little later on won the heart of a donkeyman from the Fatherland by chatting to him in German. It would be useless to attempt to detail all the cases, but all were carried away by the sympathy and charm of manner of her Majesty the Queen.

### The King Recognises Two Patients.

The second ward was next visited, and their Majesties had a word for each occupant of the beds. Two patients of particular interest were here, one of whom was Richard Green, a Londoner, who for the past year or so has been at sea as a cook. Five years ago he left the 15th Royal Hussars as a corporal after serving ten years, and when the King and Queen visited India in November 1906, Green formed one of the Royal escort at Agra, Calcutta, and Lucknow. This naturally formed a topic of interest, and the Queen recalled several incidents which Green remembered vividly. The next patient was a Maltese steward, whom the King recognised as having served in the *Thunderer* and *Dreadnought* over twenty years ago, when his Majesty was a sub-lieutenant. There was no time for a visit to be made to the *x*-ray room or to the operating theatre.

The Royal Hamadryad Hospital, Cardiff.

A few words with regard to the institution and its new buildings should be added. The new buildings are the result of a movement for celebrating in Cardiff the sixtieth year of the reign of Queen Victoria. The greater portion of the site was the gift of the late Lord Bute, while the Taff Vale Railway Company gave the valuable piece of land upon which the front of the building now stands. There are three wards, containing in all fifty-four beds, and the great utility, or perhaps it should be said the absolute necessity, of there being such a hospital is proved by the fact that it is taxed to its full capacity always, except for a few beds which are kept for emergencies. On the day when their Majesties visited there were forty-six beds in occupation.

## Figures and Tables

**Figure f1:**